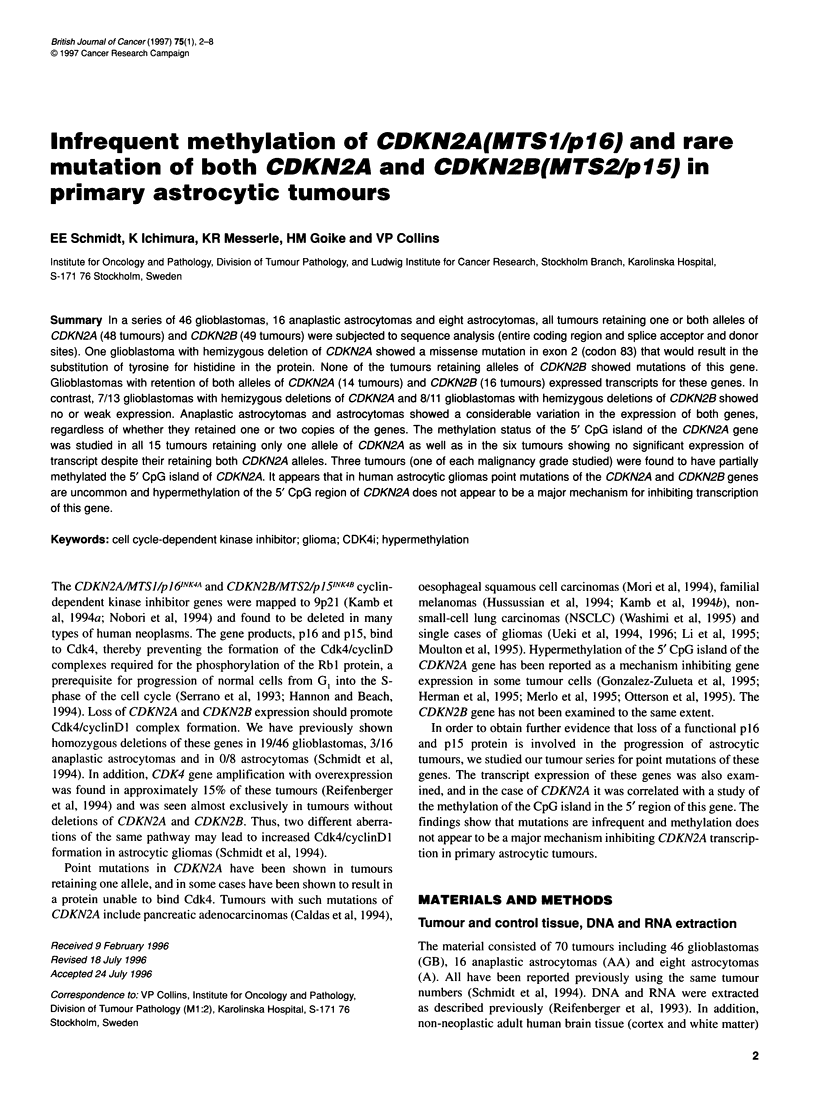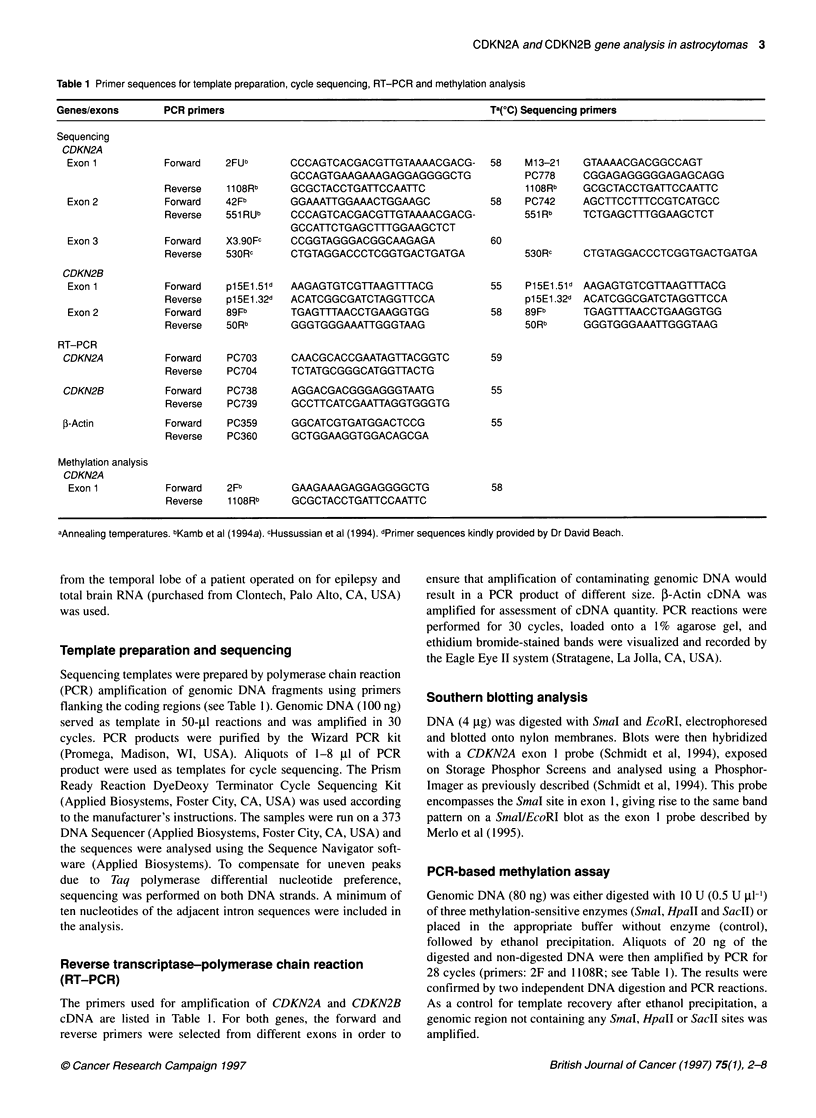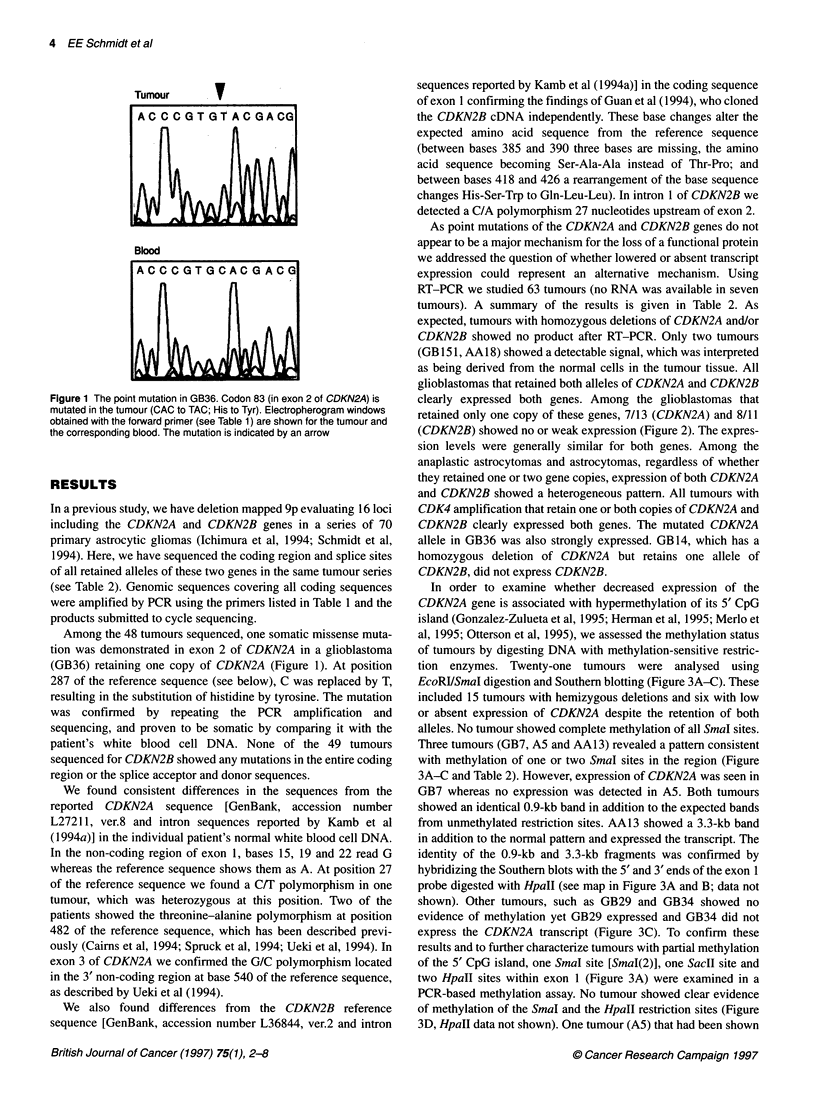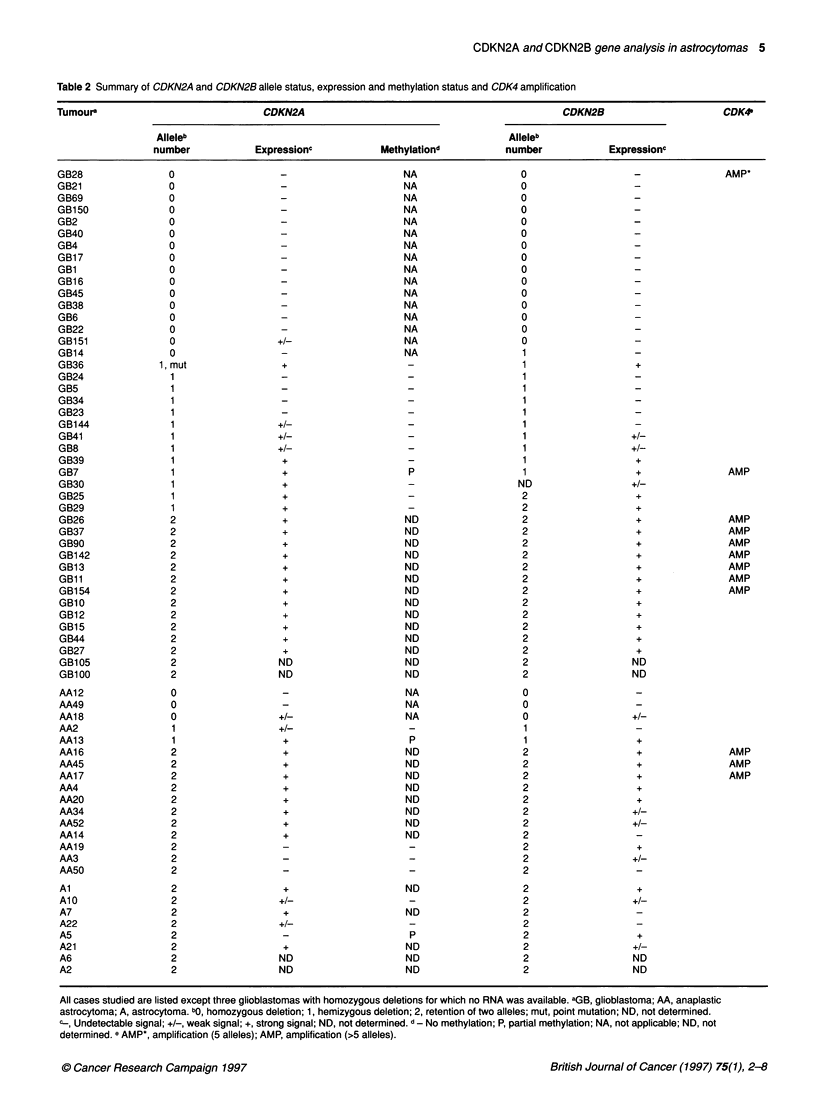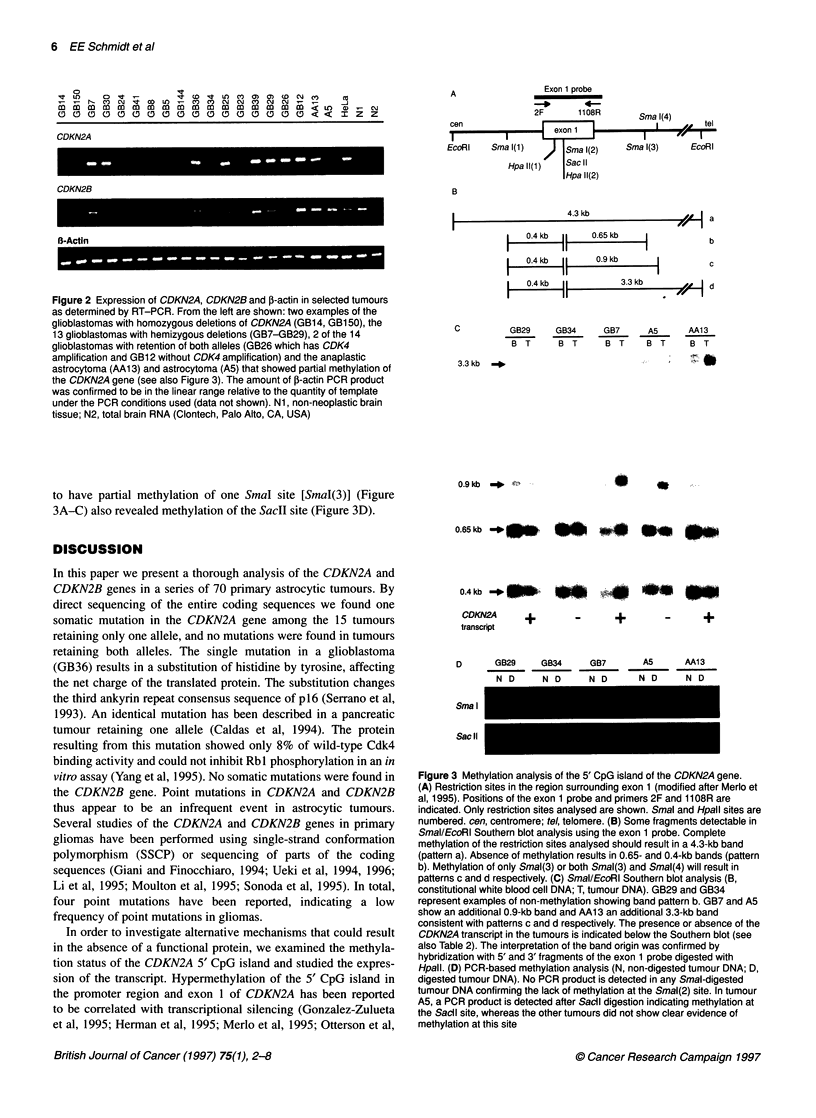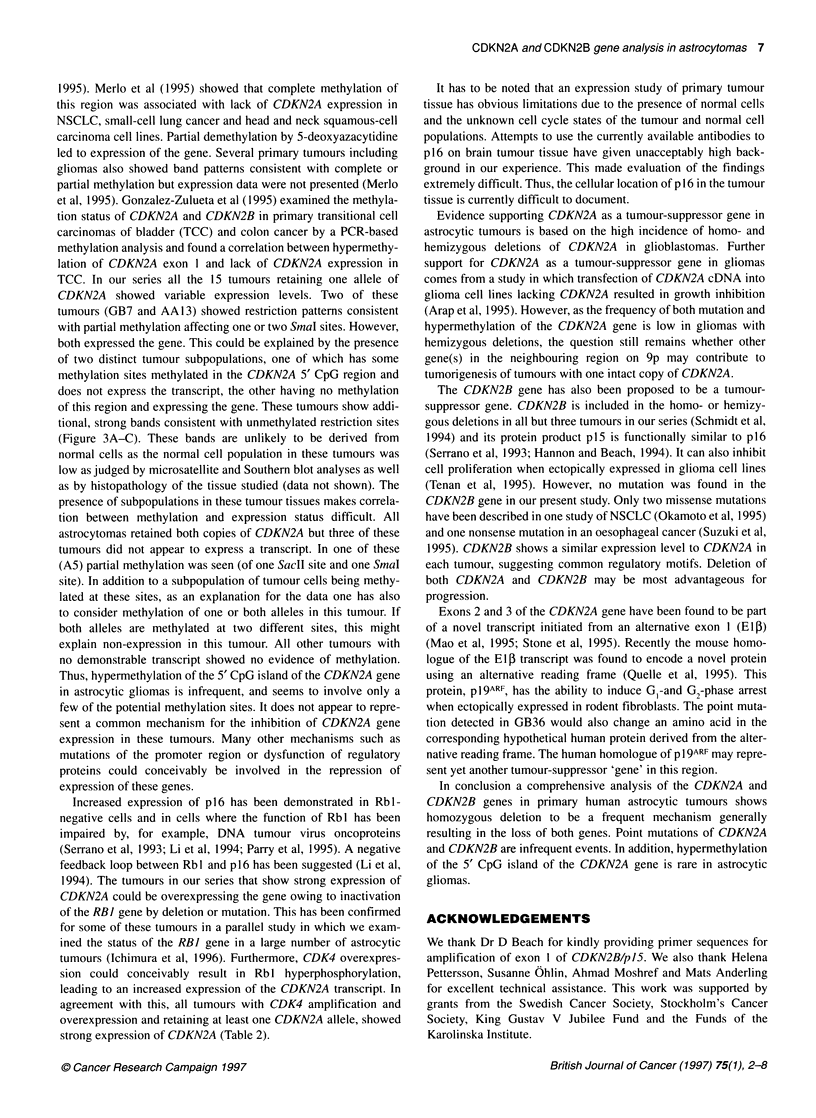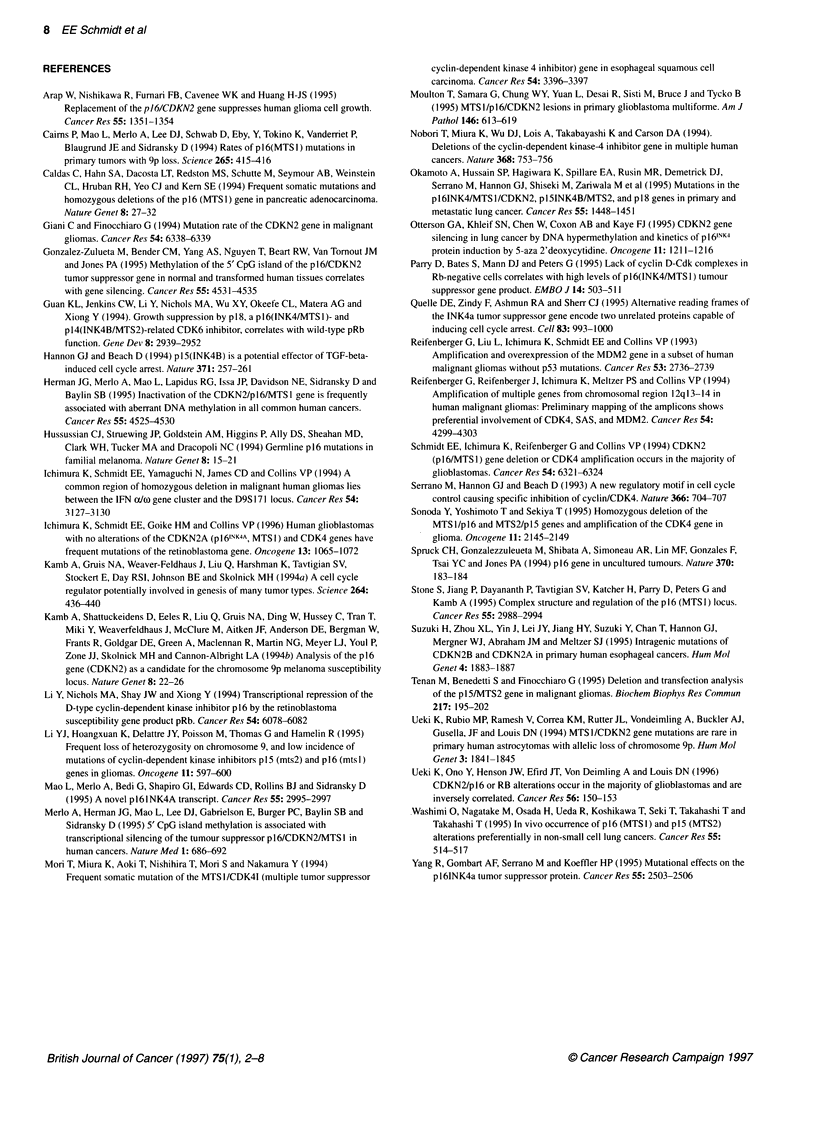# Infrequent methylation of CDKN2A(MTS1/p16) and rare mutation of both CDKN2A and CDKN2B(MTS2/p15) in primary astrocytic tumours.

**DOI:** 10.1038/bjc.1997.2

**Published:** 1997

**Authors:** E. E. Schmidt, K. Ichimura, K. R. Messerle, H. M. Goike, V. P. Collins

**Affiliations:** Institute for Oncology and Pathology, Division of Tumour Pathology, Karolinska Hospital, Stockholm, Sweden.

## Abstract

**Images:**


*British Journal of Cancer* (1997) **75**(1), 2–8
© 1997 Cancer Research Campaign

### Infrequent methylation of *CDKN2A(MTS1/p16)* and rare mutation of both *CDKN2A* and *CDKN2B(MTS2/p15)* in primary astrocytic tumours

**EE Schmidt, K Ichimura, KR Messerle, HM Goike and VP Collins**

Institute for Oncology and Pathology, Division of Tumour Pathology, and Ludwig Institute for Cancer Research, Stockholm Branch, Karolinska Hospital, S-171 76 Stockholm, Sweden

**Summary** In a series of 46 glioblastomas, 16 anaplastic astrocytomas and eight astrocytomas, all tumours retaining one or both alleles of *CDKN2A* (48 tumours) and *CDKN2B* (49 tumours) were subjected to sequence analysis (entire coding region and splice acceptor and donor sites). One glioblastoma with hemizygous deletion of *CDKN2A* showed a missense mutation in exon 2 (codon 83) that would result in the substitution of tyrosine for histidine in the protein. None of the tumours retaining alleles of *CDKN2B* showed mutations of this gene. Glioblastomas with retention of both alleles of *CDKN2A* (14 tumours) and *CDKN2B* (16 tumours) expressed transcripts for these genes. In contrast, 7/13 glioblastomas with hemizygous deletions of *CDKN2A* and 8/11 glioblastomas with hemizygous deletions of *CDKN2B* showed no or weak expression. Anaplastic astrocytomas and astrocytomas showed a considerable variation in the expression of both genes, regardless of whether they retained one or two copies of the genes. The methylation status of the 5′ CpG island of the *CDKN2A* gene was studied in all 15 tumours retaining only one allele of *CDKN2A* as well as in the six tumours showing no significant expression of transcript despite their retaining both *CDKN2A* alleles. Three tumours (one of each malignancy grade studied) were found to have partially methylated the 5′ CpG island of *CDKN2A*. It appears that in human astrocytic gliomas point mutations of the *CDKN2A* and *CDKN2B* genes are uncommon and hypermethylation of the 5′ CpG region of *CDKN2A* does not appear to be a major mechanism for inhibiting transcription of this gene.

**Keywords:** cell cycle-dependent kinase inhibitor; glioma; CDK4i; hypermethylation

The *CDKN2A/MTS1/p16*$^{INK4A}$ and *CDKN2B/MTS2/p15*$^{INK4B}$ cyclin-dependent kinase inhibitor genes were mapped to 9p21 (Kamb et al, 1994*a*; Nobori et al, 1994) and found to be deleted in many types of human neoplasms. The gene products, p16 and p15, bind to Cdk4, thereby preventing the formation of the Cdk4/cyclinD complexes required for the phosphorylation of the Rb1 protein, a prerequisite for progression of normal cells from $G_1$ into the S-phase of the cell cycle (Serrano et al, 1993; Hannon and Beach, 1994). Loss of *CDKN2A* and *CDKN2B* expression should promote Cdk4/cyclinD1 complex formation. We have previously shown homozygous deletions of these genes in 19/46 glioblastomas, 3/16 anaplastic astrocytomas and in 0/8 astrocytomas (Schmidt et al, 1994). In addition, *CDK4* gene amplification with overexpression was found in approximately 15% of these tumours (Reifenberger et al, 1994) and was seen almost exclusively in tumours without deletions of *CDKN2A* and *CDKN2B*. Thus, two different aberrations of the same pathway may lead to increased Cdk4/cyclinD1 formation in astrocytic gliomas (Schmidt et al, 1994).

Point mutations in *CDKN2A* have been shown in tumours retaining one allele, and in some cases have been shown to result in a protein unable to bind Cdk4. Tumours with such mutations of *CDKN2A* include pancreatic adenocarcinomas (Caldas et al, 1994), oesophageal squamous cell carcinomas (Mori et al, 1994), familial melanomas (Hussussian et al, 1994; Kamb et al, 1994*b*), non-small-cell lung carcinomas (NSCLC) (Washimi et al, 1995) and single cases of gliomas (Ueki et al, 1994, 1996; Li et al, 1995; Moulton et al, 1995). Hypermethylation of the 5′ CpG island of the *CDKN2A* gene has been reported as a mechanism inhibiting gene expression in some tumour cells (Gonzalez-Zulueta et al, 1995; Herman et al, 1995; Merlo et al, 1995; Otterson et al, 1995). The *CDKN2B* gene has not been examined to the same extent.

In order to obtain further evidence that loss of a functional p16 and p15 protein is involved in the progression of astrocytic tumours, we studied our tumour series for point mutations of these genes. The transcript expression of these genes was also examined, and in the case of *CDKN2A* it was correlated with a study of the methylation of the CpG island in the 5′ region of this gene. The findings show that mutations are infrequent and methylation does not appear to be a major mechanism inhibiting *CDKN2A* transcription in primary astrocytic tumours.

### MATERIALS AND METHODS

### Tumour and control tissue, DNA and RNA extraction

The material consisted of 70 tumours including 46 glioblastomas (GB), 16 anaplastic astrocytomas (AA) and eight astrocytomas (A). All have been reported previously using the same tumour numbers (Schmidt et al, 1994). DNA and RNA were extracted as described previously (Reifenberger et al, 1993). In addition, non-neoplastic adult human brain tissue (cortex and white matter)

*Received 9 February 1996*
*Revised 18 July 1996*
*Accepted 24 July 1996*

*Correspondence to:* VP Collins, Institute for Oncology and Pathology, Division of Tumour Pathology (M1:2), Karolinska Hospital, S-171 76 Stockholm, Sweden

**Table 1** Primer sequences for template preparation, cycle sequencing, RT–PCR and methylation analysis

| Genes/exons | PCR primers | | | T[a](°C) | Sequencing primers | |
|---|---|---|---|---|---|---|
| Sequencing | | | | | | |
| *CDKN2A* | | | | | | |
| Exon 1 | Forward | 2FU[b] | CCCAGTCACGACGTTGTAAAACGACG-GCCAGTGAAGAAAGAGGAGGGGCTG | 58 | M13–21<br>PC778 | GTAAAACGACGGCCAGT<br>CGGAGAGGGGGAGAGCAGG |
| | Reverse | 1108R[b] | GCGCTACCTGATTCCAATTC | | 1108R[b] | GCGCTACCTGATTCCAATTC |
| Exon 2 | Forward | 42F[b] | GGAAATTGGAAACTGGAAGC | 58 | PC742 | AGCTTCCTTTCCGTCATGCC |
| | Reverse | 551RU[b] | CCCAGTCACGACGTTGTAAAACGACG-GCCATTCTGAGCTTTGGAAGCTCT | | 551R[b] | TCTGAGCTTTGGAAGCTCT |
| Exon 3 | Forward | X3.90F[c] | CCGGTAGGGACGGCAAGAGA | 60 | | |
| | Reverse | 530R[c] | CTGTAGGACCCTCGGTGACTGATGA | | 530R[c] | CTGTAGGACCCTCGGTGACTGATGA |
| *CDKN2B* | | | | | | |
| Exon 1 | Forward | p15E1.51[d] | AAGAGTGTCGTTAAGTTTACG | 55 | P15E1.51[d] | AAGAGTGTCGTTAAGTTTACG |
| | Reverse | p15E1.32[d] | ACATCGGCGATCTAGGTTCCA | | p15E1.32[d] | ACATCGGCGATCTAGGTTCCA |
| Exon 2 | Forward | 89F[b] | TGAGTTTAACCTGAAGGTGG | 58 | 89F[b] | TGAGTTTAACCTGAAGGTGG |
| | Reverse | 50R[b] | GGGTGGGAAATTGGGTAAG | | 50R[b] | GGGTGGGAAATTGGGTAAG |
| RT–PCR | | | | | | |
| *CDKN2A* | Forward | PC703 | CAACGCACCGAATAGTTACGGTC | 59 | | |
| | Reverse | PC704 | TCTATGCGGGCATGGTTACTG | | | |
| *CDKN2B* | Forward | PC738 | AGGACGACGGGAGGGTAATG | 55 | | |
| | Reverse | PC739 | GCCTTCATCGAATTAGGTGGGTG | | | |
| β-Actin | Forward | PC359 | GGCATCGTGATGGACTCCG | 55 | | |
| | Reverse | PC360 | GCTGGAAGGTGGACAGCGA | | | |
| Methylation analysis | | | | | | |
| *CDKN2A* | | | | | | |
| Exon 1 | Forward | 2F[b] | GAAGAAAGAGGAGGGGCTG | 58 | | |
| | Reverse | 1108R[b] | GCGCTACCTGATTCCAATTC | | | |

[a]Annealing temperatures. [b]Kamb et al (1994*a*). [c]Hussussian et al (1994). [d]Primer sequences kindly provided by Dr David Beach.

from the temporal lobe of a patient operated on for epilepsy and total brain RNA (purchased from Clontech, Palo Alto, CA, USA) was used.

### Template preparation and sequencing

Sequencing templates were prepared by polymerase chain reaction (PCR) amplification of genomic DNA fragments using primers flanking the coding regions (see Table 1). Genomic DNA (100 ng) served as template in 50-μl reactions and was amplified in 30 cycles. PCR products were purified by the Wizard PCR kit (Promega, Madison, WI, USA). Aliquots of 1–8 μl of PCR product were used as templates for cycle sequencing. The Prism Ready Reaction DyeDeoxy Terminator Cycle Sequencing Kit (Applied Biosystems, Foster City, CA, USA) was used according to the manufacturer's instructions. The samples were run on a 373 DNA Sequencer (Applied Biosystems, Foster City, CA, USA) and the sequences were analysed using the Sequence Navigator software (Applied Biosystems). To compensate for uneven peaks due to *Taq* polymerase differential nucleotide preference, sequencing was performed on both DNA strands. A minimum of ten nucleotides of the adjacent intron sequences were included in the analysis.

### Reverse transcriptase–polymerase chain reaction (RT–PCR)

The primers used for amplification of *CDKN2A* and *CDKN2B* cDNA are listed in Table 1. For both genes, the forward and reverse primers were selected from different exons in order to ensure that amplification of contaminating genomic DNA would result in a PCR product of different size. β-Actin cDNA was amplified for assessment of cDNA quantity. PCR reactions were performed for 30 cycles, loaded onto a 1% agarose gel, and ethidium bromide-stained bands were visualized and recorded by the Eagle Eye II system (Stratagene, La Jolla, CA, USA).

### Southern blotting analysis

DNA (4 μg) was digested with *Sma*I and *Eco*RI, electrophoresed and blotted onto nylon membranes. Blots were then hybridized with a *CDKN2A* exon 1 probe (Schmidt et al, 1994), exposed on Storage Phosphor Screens and analysed using a PhosphorImager as previously described (Schmidt et al, 1994). This probe encompasses the *Sma*I site in exon 1, giving rise to the same band pattern on a *Sma*I/*Eco*RI blot as the exon 1 probe described by Merlo et al (1995).

### PCR-based methylation assay

Genomic DNA (80 ng) was either digested with 10 U (0.5 U $\mu l^{-1}$) of three methylation-sensitive enzymes (*Sma*I, *Hpa*II and *Sac*II) or placed in the appropriate buffer without enzyme (control), followed by ethanol precipitation. Aliquots of 20 ng of the digested and non-digested DNA were then amplified by PCR for 28 cycles (primers: 2F and 1108R; see Table 1). The results were confirmed by two independent DNA digestion and PCR reactions. As a control for template recovery after ethanol precipitation, a genomic region not containing any *Sma*I, *Hpa*II or *Sac*II sites was amplified.

© Cancer Research Campaign 1997

**Figure 1** The point mutation in GB36. Codon 83 (in exon 2 of *CDKN2A*) is mutated in the tumour (CAC to TAC; His to Tyr). Electropherogram windows obtained with the forward primer (see Table 1) are shown for the tumour and the corresponding blood. The mutation is indicated by an arrow

### RESULTS

In a previous study, we have deletion mapped 9p evaluating 16 loci including the *CDKN2A* and *CDKN2B* genes in a series of 70 primary astrocytic gliomas (Ichimura et al, 1994; Schmidt et al, 1994). Here, we have sequenced the coding region and splice sites of all retained alleles of these two genes in the same tumour series (see Table 2). Genomic sequences covering all coding sequences were amplified by PCR using the primers listed in Table 1 and the products submitted to cycle sequencing.

Among the 48 tumours sequenced, one somatic missense mutation was demonstrated in exon 2 of *CDKN2A* in a glioblastoma (GB36) retaining one copy of *CDKN2A* (Figure 1). At position 287 of the reference sequence (see below), C was replaced by T, resulting in the substitution of histidine by tyrosine. The mutation was confirmed by repeating the PCR amplification and sequencing, and proven to be somatic by comparing it with the patient's white blood cell DNA. None of the 49 tumours sequenced for *CDKN2B* showed any mutations in the entire coding region or the splice acceptor and donor sequences.

We found consistent differences in the sequences from the reported *CDKN2A* sequence [GenBank, accession number L27211, ver.8 and intron sequences reported by Kamb et al (1994*a*)] in the individual patient's normal white blood cell DNA. In the non-coding region of exon 1, bases 15, 19 and 22 read G whereas the reference sequence shows them as A. At position 27 of the reference sequence we found a C/T polymorphism in one tumour, which was heterozygous at this position. Two of the patients showed the threonine–alanine polymorphism at position 482 of the reference sequence, which has been described previously (Cairns et al, 1994; Spruck et al, 1994; Ueki et al, 1994). In exon 3 of *CDKN2A* we confirmed the G/C polymorphism located in the 3′ non-coding region at base 540 of the reference sequence, as described by Ueki et al (1994).

We also found differences from the *CDKN2B* reference sequence [GenBank, accession number L36844, ver.2 and intron sequences reported by Kamb et al (1994a)] in the coding sequence of exon 1 confirming the findings of Guan et al (1994), who cloned the *CDKN2B* cDNA independently. These base changes alter the expected amino acid sequence from the reference sequence (between bases 385 and 390 three bases are missing, the amino acid sequence becoming Ser-Ala-Ala instead of Thr-Pro; and between bases 418 and 426 a rearrangement of the base sequence changes His-Ser-Trp to Gln-Leu-Leu). In intron 1 of *CDKN2B* we detected a C/A polymorphism 27 nucleotides upstream of exon 2.

As point mutations of the *CDKN2A* and *CDKN2B* genes do not appear to be a major mechanism for the loss of a functional protein we addressed the question of whether lowered or absent transcript expression could represent an alternative mechanism. Using RT–PCR we studied 63 tumours (no RNA was available in seven tumours). A summary of the results is given in Table 2. As expected, tumours with homozygous deletions of *CDKN2A* and/or *CDKN2B* showed no product after RT–PCR. Only two tumours (GB151, AA18) showed a detectable signal, which was interpreted as being derived from the normal cells in the tumour tissue. All glioblastomas that retained both alleles of *CDKN2A* and *CDKN2B* clearly expressed both genes. Among the glioblastomas that retained only one copy of these genes, 7/13 (*CDKN2A*) and 8/11 (*CDKN2B*) showed no or weak expression (Figure 2). The expression levels were generally similar for both genes. Among the anaplastic astrocytomas and astrocytomas, regardless of whether they retained one or two gene copies, expression of both *CDKN2A* and *CDKN2B* showed a heterogeneous pattern. All tumours with *CDK4* amplification that retain one or both copies of *CDKN2A* and *CDKN2B* clearly expressed both genes. The mutated *CDKN2A* allele in GB36 was also strongly expressed. GB14, which has a homozygous deletion of *CDKN2A* but retains one allele of *CDKN2B*, did not express *CDKN2B*.

In order to examine whether decreased expression of the *CDKN2A* gene is associated with hypermethylation of its 5′ CpG island (Gonzalez-Zulueta et al, 1995; Herman et al, 1995; Merlo et al, 1995; Otterson et al, 1995), we assessed the methylation status of tumours by digesting DNA with methylation-sensitive restriction enzymes. Twenty-one tumours were analysed using *Eco*RI/*Sma*I digestion and Southern blotting (Figure 3A–C). These included 15 tumours with hemizygous deletions and six with low or absent expression of *CDKN2A* despite the retention of both alleles. No tumour showed complete methylation of all *Sma*I sites. Three tumours (GB7, A5 and AA13) revealed a pattern consistent with methylation of one or two *Sma*I sites in the region (Figure 3A–C and Table 2). However, expression of *CDKN2A* was seen in GB7 whereas no expression was detected in A5. Both tumours showed an identical 0.9-kb band in addition to the expected bands from unmethylated restriction sites. AA13 showed a 3.3-kb band in addition to the normal pattern and expressed the transcript. The identity of the 0.9-kb and 3.3-kb fragments was confirmed by hybridizing the Southern blots with the 5′ and 3′ ends of the exon 1 probe digested with *Hpa*II (see map in Figure 3A and B; data not shown). Other tumours, such as GB29 and GB34 showed no evidence of methylation yet GB29 expressed and GB34 did not express the *CDKN2A* transcript (Figure 3C). To confirm these results and to further characterize tumours with partial methylation of the 5′ CpG island, one *Sma*I site [*Sma*I(2)], one *Sac*II site and two *Hpa*II sites within exon 1 (Figure 3A) were examined in a PCR-based methylation assay. No tumour showed clear evidence of methylation of the *Sma*I and the *Hpa*II restriction sites (Figure 3D, *Hpa*II data not shown). One tumour (A5) that had been shown

 © Cancer Research Campaign 1997

**Table 2** Summary of *CDKN2A* and *CDKN2B* allele status, expression and methylation status and *CDK4* amplification

| **Tumour**[a] | ***CDKN2A*** | | | ***CDKN2B*** | | ***CDK4***[e] |
|---|---|---|---|---|---|---|
| | **Allele**[b] **number** | **Expression**[c] | **Methylation**[d] | **Allele**[b] **number** | **Expression**[c] | |
| GB28 | 0 | – | NA | 0 | – | AMP* |
| GB21 | 0 | – | NA | 0 | – | |
| GB69 | 0 | – | NA | 0 | – | |
| GB150 | 0 | – | NA | 0 | – | |
| GB2 | 0 | – | NA | 0 | – | |
| GB40 | 0 | – | NA | 0 | – | |
| GB4 | 0 | – | NA | 0 | – | |
| GB17 | 0 | – | NA | 0 | – | |
| GB1 | 0 | – | NA | 0 | – | |
| GB16 | 0 | – | NA | 0 | – | |
| GB45 | 0 | – | NA | 0 | – | |
| GB38 | 0 | – | NA | 0 | – | |
| GB6 | 0 | – | NA | 0 | – | |
| GB22 | 0 | – | NA | 0 | – | |
| GB151 | 0 | +/– | NA | 0 | – | |
| GB14 | 0 | – | NA | 1 | – | |
| GB36 | 1, mut | + | – | 1 | + | |
| GB24 | 1 | – | – | 1 | – | |
| GB5 | 1 | – | – | 1 | – | |
| GB34 | 1 | – | – | 1 | – | |
| GB23 | 1 | – | – | 1 | – | |
| GB144 | 1 | +/– | – | 1 | – | |
| GB41 | 1 | +/– | – | 1 | +/– | |
| GB8 | 1 | +/– | – | 1 | +/– | |
| GB39 | 1 | + | – | 1 | + | |
| GB7 | 1 | + | P | 1 | + | AMP |
| GB30 | 1 | + | – | ND | +/– | |
| GB25 | 1 | + | – | 2 | + | |
| GB29 | 1 | + | – | 2 | + | |
| GB26 | 2 | + | ND | 2 | + | AMP |
| GB37 | 2 | + | ND | 2 | + | AMP |
| GB90 | 2 | + | ND | 2 | + | AMP |
| GB142 | 2 | + | ND | 2 | + | AMP |
| GB13 | 2 | + | ND | 2 | + | AMP |
| GB11 | 2 | + | ND | 2 | + | AMP |
| GB154 | 2 | + | ND | 2 | + | AMP |
| GB10 | 2 | + | ND | 2 | + | |
| GB12 | 2 | + | ND | 2 | + | |
| GB15 | 2 | + | ND | 2 | + | |
| GB44 | 2 | + | ND | 2 | + | |
| GB27 | 2 | + | ND | 2 | + | |
| GB105 | 2 | ND | ND | 2 | ND | |
| GB100 | 2 | ND | ND | 2 | ND | |
| AA12 | 0 | – | NA | 0 | – | |
| AA49 | 0 | – | NA | 0 | – | |
| AA18 | 0 | +/– | NA | 0 | +/– | |
| AA2 | 1 | +/– | – | 1 | – | |
| AA13 | 1 | + | P | 1 | + | |
| AA16 | 2 | + | ND | 2 | + | AMP |
| AA45 | 2 | + | ND | 2 | + | AMP |
| AA17 | 2 | + | ND | 2 | + | AMP |
| AA4 | 2 | + | ND | 2 | + | |
| AA20 | 2 | + | ND | 2 | + | |
| AA34 | 2 | + | ND | 2 | +/– | |
| AA52 | 2 | + | ND | 2 | +/– | |
| AA14 | 2 | + | ND | 2 | – | |
| AA19 | 2 | – | – | 2 | + | |
| AA3 | 2 | – | – | 2 | +/– | |
| AA50 | 2 | – | – | 2 | – | |
| A1 | 2 | + | ND | 2 | + | |
| A10 | 2 | +/– | – | 2 | +/– | |
| A7 | 2 | + | ND | 2 | – | |
| A22 | 2 | +/– | – | 2 | – | |
| A5 | 2 | – | P | 2 | + | |
| A21 | 2 | + | ND | 2 | +/– | |
| A6 | 2 | ND | ND | 2 | ND | |
| A2 | 2 | ND | ND | 2 | ND | |

All cases studied are listed except three glioblastomas with homozygous deletions for which no RNA was available. [a]GB, glioblastoma; AA, anaplastic astrocytoma; A, astrocytoma. [b]0, homozygous deletion; 1, hemizygous deletion; 2, retention of two alleles; mut, point mutation; ND, not determined. [c]–, Undetectable signal; +/–, weak signal; +, strong signal; ND, not determined. [d] – No methylation; P, partial methylation; NA, not applicable; ND, not determined. [e] AMP*, amplification (5 alleles); AMP, amplification (>5 alleles).

*© Cancer Research Campaign 1997* 

**Figure 2** Expression of *CDKN2A*, *CDKN2B* and β-actin in selected tumours as determined by RT–PCR. From the left are shown: two examples of the glioblastomas with homozygous deletions of *CDKN2A* (GB14, GB150), the 13 glioblastomas with hemizygous deletions (GB7–GB29), 2 of the 14 glioblastomas with retention of both alleles (GB26 which has *CDK4* amplification and GB12 without *CDK4* amplification) and the anaplastic astrocytoma (AA13) and astrocytoma (A5) that showed partial methylation of the *CDKN2A* gene (see also Figure 3). The amount of β-actin PCR product was confirmed to be in the linear range relative to the quantity of template under the PCR conditions used (data not shown). N1, non-neoplastic brain tissue; N2, total brain RNA (Clontech, Palo Alto, CA, USA)

to have partial methylation of one *Sma*I site [*Sma*I(3)] (Figure 3A–C) also revealed methylation of the *Sac*II site (Figure 3D).

### DISCUSSION

In this paper we present a thorough analysis of the *CDKN2A* and *CDKN2B* genes in a series of 70 primary astrocytic tumours. By direct sequencing of the entire coding sequences we found one somatic mutation in the *CDKN2A* gene among the 15 tumours retaining only one allele, and no mutations were found in tumours retaining both alleles. The single mutation in a glioblastoma (GB36) results in a substitution of histidine by tyrosine, affecting the net charge of the translated protein. The substitution changes the third ankyrin repeat consensus sequence of p16 (Serrano et al, 1993). An identical mutation has been described in a pancreatic tumour retaining one allele (Caldas et al, 1994). The protein resulting from this mutation showed only 8% of wild-type Cdk4 binding activity and could not inhibit Rb1 phosphorylation in an *in vitro* assay (Yang et al, 1995). No somatic mutations were found in the *CDKN2B* gene. Point mutations in *CDKN2A* and *CDKN2B* thus appear to be an infrequent event in astrocytic tumours. Several studies of the *CDKN2A* and *CDKN2B* genes in primary gliomas have been performed using single-strand conformation polymorphism (SSCP) or sequencing of parts of the coding sequences (Giani and Finocchiaro, 1994; Ueki et al, 1994, 1996; Li et al, 1995; Moulton et al, 1995; Sonoda et al, 1995). In total, four point mutations have been reported, indicating a low frequency of point mutations in gliomas.

In order to investigate alternative mechanisms that could result in the absence of a functional protein, we examined the methylation status of the *CDKN2A* 5′ CpG island and studied the expression of the transcript. Hypermethylation of the 5′ CpG island in the promoter region and exon 1 of *CDKN2A* has been reported to be correlated with transcriptional silencing (Gonzalez-Zulueta et al, 1995; Herman et al, 1995; Merlo et al, 1995; Otterson et al,

**Figure 3** Methylation analysis of the 5′ CpG island of the *CDKN2A* gene. (**A**) Restriction sites in the region surrounding exon 1 (modified after Merlo et al, 1995). Positions of the exon 1 probe and primers 2F and 1108R are indicated. Only restriction sites analysed are shown. *Sma*I and *Hpa*II sites are numbered. *cen*, centromere; *tel*, telomere. (**B**) Some fragments detectable in *Sma*I/*Eco*RI Southern blot analysis using the exon 1 probe. Complete methylation of the restriction sites analysed should result in a 4.3-kb band (pattern a). Absence of methylation results in 0.65- and 0.4-kb bands (pattern b). Methylation of only *Sma*I(3) or both *Sma*I(3) and *Sma*I(4) will result in patterns c and d respectively. (**C**) *Sma*I/*Eco*RI Southern blot analysis (B, constitutional white blood cell DNA; T, tumour DNA). GB29 and GB34 represent examples of non-methylation showing band pattern b. GB7 and A5 show an additional 0.9-kb band and AA13 an additional 3.3-kb band consistent with patterns c and d respectively. The presence or absence of the *CDKN2A* transcript in the tumours is indicated below the Southern blot (see also Table 2). The interpretation of the band origin was confirmed by hybridization with 5′ and 3′ fragments of the exon 1 probe digested with *Hpa*II. (**D**) PCR-based methylation analysis (N, non-digested tumour DNA; D, digested tumour DNA). No PCR product is detected in any *Sma*I-digested tumour DNA confirming the lack of methylation at the *Sma*I(2) site. In tumour A5, a PCR product is detected after *Sac*II digestion indicating methylation at the *Sac*II site, whereas the other tumours did not show clear evidence of methylation at this site

© Cancer Research Campaign 1997

1995). Merlo et al (1995) showed that complete methylation of this region was associated with lack of *CDKN2A* expression in NSCLC, small-cell lung cancer and head and neck squamous-cell carcinoma cell lines. Partial demethylation by 5-deoxyazacytidine led to expression of the gene. Several primary tumours including gliomas also showed band patterns consistent with complete or partial methylation but expression data were not presented (Merlo et al, 1995). Gonzalez-Zulueta et al (1995) examined the methylation status of *CDKN2A* and *CDKN2B* in primary transitional cell carcinomas of bladder (TCC) and colon cancer by a PCR-based methylation analysis and found a correlation between hypermethylation of *CDKN2A* exon 1 and lack of *CDKN2A* expression in TCC. In our series all the 15 tumours retaining one allele of *CDKN2A* showed variable expression levels. Two of these tumours (GB7 and AA13) showed restriction patterns consistent with partial methylation affecting one or two *Sma*I sites. However, both expressed the gene. This could be explained by the presence of two distinct tumour subpopulations, one of which has some methylation sites methylated in the *CDKN2A* 5′ CpG region and does not express the transcript, the other having no methylation of this region and expressing the gene. These tumours show additional, strong bands consistent with unmethylated restriction sites (Figure 3A–C). These bands are unlikely to be derived from normal cells as the normal cell population in these tumours was low as judged by microsatellite and Southern blot analyses as well as by histopathology of the tissue studied (data not shown). The presence of subpopulations in these tumour tissues makes correlation between methylation and expression status difficult. All astrocytomas retained both copies of *CDKN2A* but three of these tumours did not appear to express a transcript. In one of these (A5) partial methylation was seen (of one *Sac*II site and one *Sma*I site). In addition to a subpopulation of tumour cells being methylated at these sites, as an explanation for the data one has also to consider methylation of one or both alleles in this tumour. If both alleles are methylated at two different sites, this might explain non-expression in this tumour. All other tumours with no demonstrable transcript showed no evidence of methylation. Thus, hypermethylation of the 5′ CpG island of the *CDKN2A* gene in astrocytic gliomas is infrequent, and seems to involve only a few of the potential methylation sites. It does not appear to represent a common mechanism for the inhibition of *CDKN2A* gene expression in these tumours. Many other mechanisms such as mutations of the promoter region or dysfunction of regulatory proteins could conceivably be involved in the repression of expression of these genes.

Increased expression of p16 has been demonstrated in Rb1-negative cells and in cells where the function of Rb1 has been impaired by, for example, DNA tumour virus oncoproteins (Serrano et al, 1993; Li et al, 1994; Parry et al, 1995). A negative feedback loop between Rb1 and p16 has been suggested (Li et al, 1994). The tumours in our series that show strong expression of *CDKN2A* could be overexpressing the gene owing to inactivation of the *RB1* gene by deletion or mutation. This has been confirmed for some of these tumours in a parallel study in which we examined the status of the *RB1* gene in a large number of astrocytic tumours (Ichimura et al, 1996). Furthermore, *CDK4* overexpression could conceivably result in Rb1 hyperphosphorylation, leading to an increased expression of the *CDKN2A* transcript. In agreement with this, all tumours with *CDK4* amplification and overexpression and retaining at least one *CDKN2A* allele, showed strong expression of *CDKN2A* (Table 2).

It has to be noted that an expression study of primary tumour tissue has obvious limitations due to the presence of normal cells and the unknown cell cycle states of the tumour and normal cell populations. Attempts to use the currently available antibodies to p16 on brain tumour tissue have given unacceptably high background in our experience. This made evaluation of the findings extremely difficult. Thus, the cellular location of p16 in the tumour tissue is currently difficult to document.

Evidence supporting *CDKN2A* as a tumour-suppressor gene in astrocytic tumours is based on the high incidence of homo- and hemizygous deletions of *CDKN2A* in glioblastomas. Further support for *CDKN2A* as a tumour-suppressor gene in gliomas comes from a study in which transfection of *CDKN2A* cDNA into glioma cell lines lacking *CDKN2A* resulted in growth inhibition (Arap et al, 1995). However, as the frequency of both mutation and hypermethylation of the *CDKN2A* gene is low in gliomas with hemizygous deletions, the question still remains whether other gene(s) in the neighbouring region on 9p may contribute to tumorigenesis of tumours with one intact copy of *CDKN2A*.

The *CDKN2B* gene has also been proposed to be a tumour-suppressor gene. *CDKN2B* is included in the homo- or hemizygous deletions in all but three tumours in our series (Schmidt et al, 1994) and its protein product p15 is functionally similar to p16 (Serrano et al, 1993; Hannon and Beach, 1994). It can also inhibit cell proliferation when ectopically expressed in glioma cell lines (Tenan et al, 1995). However, no mutation was found in the *CDKN2B* gene in our present study. Only two missense mutations have been described in one study of NSCLC (Okamoto et al, 1995) and one nonsense mutation in an oesophageal cancer (Suzuki et al, 1995). *CDKN2B* shows a similar expression level to *CDKN2A* in each tumour, suggesting common regulatory motifs. Deletion of both *CDKN2A* and *CDKN2B* may be most advantageous for progression.

Exons 2 and 3 of the *CDKN2A* gene have been found to be part of a novel transcript initiated from an alternative exon 1 (E1β) (Mao et al, 1995; Stone et al, 1995). Recently the mouse homologue of the E1β transcript was found to encode a novel protein using an alternative reading frame (Quelle et al, 1995). This protein, $p19^{ARF}$, has the ability to induce $G_1$-and $G_2$-phase arrest when ectopically expressed in rodent fibroblasts. The point mutation detected in GB36 would also change an amino acid in the corresponding hypothetical human protein derived from the alternative reading frame. The human homologue of $p19^{ARF}$ may represent yet another tumour-suppressor 'gene' in this region.

In conclusion a comprehensive analysis of the *CDKN2A* and *CDKN2B* genes in primary human astrocytic tumours shows homozygous deletion to be a frequent mechanism generally resulting in the loss of both genes. Point mutations of *CDKN2A* and *CDKN2B* are infrequent events. In addition, hypermethylation of the 5′ CpG island of the *CDKN2A* gene is rare in astrocytic gliomas.

### ACKNOWLEDGEMENTS

We thank Dr D Beach for kindly providing primer sequences for amplification of exon 1 of *CDKN2B/p15*. We also thank Helena Pettersson, Susanne Öhlin, Ahmad Moshref and Mats Anderling for excellent technical assistance. This work was supported by grants from the Swedish Cancer Society, Stockholm's Cancer Society, King Gustav V Jubilee Fund and the Funds of the Karolinska Institute.

© Cancer Research Campaign 1997

### REFERENCES

Arap W, Nishikawa R, Furnari FB, Cavenee WK and Huang H-JS (1995) Replacement of the *p16/CDKN2* gene suppresses human glioma cell growth. *Cancer Res* **55:** 1351–1354

Cairns P, Mao L, Merlo A, Lee DJ, Schwab D, Eby, Y, Tokino K, Vanderriet P, Blaugrund JE and Sidransky D (1994) Rates of p16(MTS1) mutations in primary tumors with 9p loss. *Science* **265:** 415–416

Caldas C, Hahn SA, Dacosta LT, Redston MS, Schutte M, Seymour AB, Weinstein CL, Hruban RH, Yeo CJ and Kern SE (1994) Frequent somatic mutations and homozygous deletions of the p16 (MTS1) gene in pancreatic adenocarcinoma. *Nature Genet* **8:** 27–32

Giani C and Finocchiaro G (1994) Mutation rate of the CDKN2 gene in malignant gliomas. *Cancer Res* **54:** 6338–6339

Gonzalez-Zulueta M, Bender CM, Yang AS, Nguyen T, Beart RW, Van Tornout JM and Jones PA (1995) Methylation of the 5′ CpG island of the p16/CDKN2 tumor suppressor gene in normal and transformed human tissues correlates with gene silencing. *Cancer Res* **55:** 4531–4535

Guan KL, Jenkins CW, Li Y, Nichols MA, Wu XY, Okeefe CL, Matera AG and Xiong Y (1994). Growth suppression by p18, a p16(INK4/MTS1)- and p14(INK4B/MTS2)-related CDK6 inhibitor, correlates with wild-type pRb function. *Gene Dev* **8:** 2939–2952

Hannon GJ and Beach D (1994) p15(INK4B) is a potential effector of TGF-beta-induced cell cycle arrest. *Nature* **371:** 257–261

Herman JG, Merlo A, Mao L, Lapidus RG, Issa JP, Davidson NE, Sidransky D and Baylin SB (1995) Inactivation of the CDKN2/p16/MTS1 gene is frequently associated with aberrant DNA methylation in all common human cancers. *Cancer Res* **55:** 4525–4530

Hussussian CJ, Struewing JP, Goldstein AM, Higgins P, Ally DS, Sheahan MD, Clark WH, Tucker MA and Dracopoli NC (1994) Germline p16 mutations in familial melanoma. *Nature Genet* **8:** 15–21

Ichimura K, Schmidt EE, Yamaguchi N, James CD and Collins VP (1994) A common region of homozygous deletion in malignant human gliomas lies between the IFN α/ω gene cluster and the D9S171 locus. *Cancer Res* **54:** 3127–3130

Ichimura K, Schmidt EE, Goike HM and Collins VP (1996) Human glioblastomas with no alterations of the CDKN2A ($p16^{INK4A}$, MTS1) and CDK4 genes have frequent mutations of the retinoblastoma gene. *Oncogene* **13:** 1065–1072

Kamb A, Gruis NA, Weaver-Feldhaus J, Liu Q, Harshman K, Tavtigian SV, Stockert E, Day RSI, Johnson BE and Skolnick MH (1994*a*) A cell cycle regulator potentially involved in genesis of many tumor types. *Science* **264:** 436–440

Kamb A, Shattuckeidens D, Eeles R, Liu Q, Gruis NA, Ding W, Hussey C, Tran T, Miki Y, Weaverfeldhaus J, McClure M, Aitken JF, Anderson DE, Bergman W, Frants R, Goldgar DE, Green A, Maclennan R, Martin NG, Meyer LJ, Youl P, Zone JJ, Skolnick MH and Cannon-Albright LA (1994*b*) Analysis of the p16 gene (CDKN2) as a candidate for the chromosome 9p melanoma susceptibility locus. *Nature Genet* **8:** 22–26

Li Y, Nichols MA, Shay JW and Xiong Y (1994) Transcriptional repression of the D-type cyclin-dependent kinase inhibitor p16 by the retinoblastoma susceptibility gene product pRb. *Cancer Res* **54:** 6078–6082

Li YJ, Hoangxuan K, Delattre JY, Poisson M, Thomas G and Hamelin R (1995) Frequent loss of heterozygosity on chromosome 9, and low incidence of mutations of cyclin-dependent kinase inhibitors p15 (mts2) and p16 (mts1) genes in gliomas. *Oncogene* **11:** 597–600

Mao L, Merlo A, Bedi G, Shapiro GI, Edwards CD, Rollins BJ and Sidransky D (1995) A novel p16INK4A transcript. *Cancer Res* **55:** 2995–2997

Merlo A, Herman JG, Mao L, Lee DJ, Gabrielson E, Burger PC, Baylin SB and Sidransky D (1995) 5′ CpG island methylation is associated with transcriptional silencing of the tumour suppressor p16/CDKN2/MTS1 in human cancers. *Nature Med* **1:** 686–692

Mori T, Miura K, Aoki T, Nishihira T, Mori S and Nakamura Y (1994) Frequent somatic mutation of the MTS1/CDK4I (multiple tumor suppressor cyclin-dependent kinase 4 inhibitor) gene in esophageal squamous cell carcinoma. *Cancer Res* **54:** 3396–3397

Moulton T, Samara G, Chung WY, Yuan L, Desai R, Sisti M, Bruce J and Tycko B (1995) MTS1/p16/CDKN2 lesions in primary glioblastoma multiforme. *Am J Pathol* **146:** 613–619

Nobori T, Miura K, Wu DJ, Lois A, Takabayashi K and Carson DA (1994). Deletions of the cyclin-dependent kinase-4 inhibitor gene in multiple human cancers. *Nature* **368:** 753–756

Okamoto A, Hussain SP, Hagiwara K, Spillare EA, Rusin MR, Demetrick DJ, Serrano M, Hannon GJ, Shiseki M, Zariwala M et al (1995) Mutations in the p16INK4/MTS1/CDKN2, p15INK4B/MTS2, and p18 genes in primary and metastatic lung cancer. *Cancer Res* **55:** 1448–1451

Otterson GA, Khleif SN, Chen W, Coxon AB and Kaye FJ (1995) CDKN2 gene silencing in lung cancer by DNA hypermethylation and kinetics of $p16^{INK4}$ protein induction by 5-aza 2′deoxycytidine. *Oncogene* **11:** 1211–1216

Parry D, Bates S, Mann DJ and Peters G (1995) Lack of cyclin D-Cdk complexes in Rb-negative cells correlates with high levels of p16(INK4/MTS1) tumour suppressor gene product. *EMBO J* **14:** 503–511

Quelle DE, Zindy F, Ashmun RA and Sherr CJ (1995) Alternative reading frames of the INK4a tumor suppressor gene encode two unrelated proteins capable of inducing cell cycle arrest. *Cell* **83:** 993–1000

Reifenberger G, Liu L, Ichimura K, Schmidt EE and Collins VP (1993) Amplification and overexpression of the MDM2 gene in a subset of human malignant gliomas without p53 mutations. *Cancer Res* **53:** 2736–2739

Reifenberger G, Reifenberger J, Ichimura K, Meltzer PS and Collins VP (1994) Amplification of multiple genes from chromosomal region 12q13–14 in human malignant gliomas: Preliminary mapping of the amplicons shows preferential involvement of CDK4, SAS, and MDM2. *Cancer Res* **54:** 4299–4303

Schmidt EE, Ichimura K, Reifenberger G and Collins VP (1994) CDKN2 (p16/MTS1) gene deletion or CDK4 amplification occurs in the majority of glioblastomas. *Cancer Res* **54:** 6321–6324

Serrano M, Hannon GJ and Beach D (1993) A new regulatory motif in cell cycle control causing specific inhibition of cyclin/CDK4. *Nature* **366:** 704–707

Sonoda Y, Yoshimoto T and Sekiya T (1995) Homozygous deletion of the MTS1/p16 and MTS2/p15 genes and amplification of the CDK4 gene in glioma. *Oncogene* **11:** 2145–2149

Spruck CH, Gonzalezzuleueta M, Shibata A, Simoneau AR, Lin MF, Gonzales F, Tsai YC and Jones PA (1994) p16 gene in uncultured tumours. *Nature* **370:** 183–184

Stone S, Jiang P, Dayananth P, Tavtigian SV, Katcher H, Parry D, Peters G and Kamb A (1995) Complex structure and regulation of the p16 (MTS1) locus. *Cancer Res* **55:** 2988–2994

Suzuki H, Zhou XL, Yin J, Lei JY, Jiang HY, Suzuki Y, Chan T, Hannon GJ, Mergner WJ, Abraham JM and Meltzer SJ (1995) Intragenic mutations of CDKN2B and CDKN2A in primary human esophageal cancers. *Hum Mol Genet* **4:** 1883–1887

Tenan M, Benedetti S and Finocchiaro G (1995) Deletion and transfection analysis of the p15/MTS2 gene in malignant gliomas. *Biochem Biophys Res Commun* **217:** 195–202

Ueki K, Rubio MP, Ramesh V, Correa KM, Rutter JL, Vondeimling A, Buckler AJ, Gusella, JF and Louis DN (1994) MTS1/CDKN2 gene mutations are rare in primary human astrocytomas with allelic loss of chromosome 9p. *Hum Mol Genet* **3:** 1841–1845

Ueki K, Ono Y, Henson JW, Efird JT, Von Deimling A and Louis DN (1996) CDKN2/p16 or RB alterations occur in the majority of glioblastomas and are inversely correlated. *Cancer Res* **56:** 150–153

Washimi O, Nagatake M, Osada H, Ueda R, Koshikawa T, Seki T, Takahashi T and Takahashi T (1995) In vivo occurrence of p16 (MTS1) and p15 (MTS2) alterations preferentially in non-small cell lung cancers. *Cancer Res* **55:** 514–517

Yang R, Gombart AF, Serrano M and Koeffler HP (1995) Mutational effects on the p16INK4a tumor suppressor protein. *Cancer Res* **55:** 2503–2506

© Cancer Research Campaign 1997